# Left ventricular ejection fraction assessed by echocardiography and magnetic resonance imaging in acute anterior and non-anterior STEMI

**DOI:** 10.1186/1532-429X-17-S1-P159

**Published:** 2015-02-03

**Authors:** Gert Klug, Sebastian J Reinstadler, Hans-Josef Feistritzer, Bastian Pernter, Wolfgang-Michael Franz, Silvana Müller, Bernhard Metzler

**Affiliations:** University Clinic of Internal Medicine III, Cardiology and Angiology, Medical University Innsbruck, Innsbruck, Austria

## Background

We have previously shown a moderate agreement of echocardiography and cardiac magnetic resonance (CMR) in the assessment of left ventricular ejection fraction (LVEF) after acute ST-segment elevation infarction (STEMI). As ejection fraction after STEMI is of paramount prognostic importance we investigated the impact of infarct location on the agreement of echocardiography and CMR.

## Methods

One-hundred and seventy-nine patients (mean age: 57 ± 11 years, n= 27 female) with first acute STEMI were enrolled in this single-center registry study. Patients underwent CMR (median: 2.4 days) and echocardiography (median 3 days) within the first week after admission. LVEF was determined from short-axis slices with CMR and with a modified Simpson rule from apical 4-chamber echo views. Infarct size was determined from late-gadolinium enhanced (LGE) CMR.

## Results

Mean LVEF determined by echocardiography was 50 ± 10% and 52 ± 11% as determined by CMR (paired Wilcoxon test: p = 0.021). The correlation between echocardiography and CMR was moderate (r: 0.492, p<0.001). The correlation of LVEF with infarct size was r:-0.306 (echocardiography) and r:-0.430 (CMR) respectively (both p<0.001). The agreement of echocardiography and CMR was higher in anterior STEMI (n=73) (r: 0.629, p<0.001) compared to non-anterior STEMI (n=105) (r: 0.279, p=0.004) (z-score: 2.92, p=0.003). The correlation of both methods with infract size was higher in anterior STEMI (CMR r:-0.588 and echocardiography r:-0.485, both p<0.001) than in non-anterior STEMI (CMR r:-0.293, p<0.001 and echo r:-0.075, p=NS) (z-scores -2.4 and -3.0, both p<0.02).

## Conclusions

LVEF by CMR is higher than estimated by echocardiography. The agreement of both methods is significantly higher in anterior STEMI than in non-anterior STEMI. Interestingly both methods show only weak to non-significant correlations of LVEF and infarct size in non-anterior STEMI. These results might help to select patients for CMR prior to therapeutic decisions based on LVEF after acute STEMI.

## Funding

Austrian Society of Cardiology, MUI-Start.

